# Investigation of Avian Reovirus Evolution and Cross-Species Transmission in Turkey Hosts by Segment-Based Temporal Analysis

**DOI:** 10.3390/v17070926

**Published:** 2025-06-28

**Authors:** Cheng-Shun Hsueh, Michael Zeller, Amro Hashish, Olufemi Fasina, Pablo Piñeyro, Ganwu Li, Jianqiang Zhang, Mohamed El-Gazzar, Yuko Sato

**Affiliations:** 1 Department of Veterinary Pathology, College of Veterinary Medicine, Iowa State University, Ames, IA 50011, USA; hsueh@iastate.edu (C.-S.H.); ofasina1@iastate.edu (O.F.); 2 Department of Veterinary Diagnostic and Production Animal Medicine, College of Veterinary Medicine, Iowa State University, Ames, IA 50011, USA; hashish@iastate.edu (A.H.); pablop@iastate.edu (P.P.); liganwu@iastate.edu (G.L.); jqzhang@iastate.edu (J.Z.); elgazzar@iastate.edu (M.E.-G.)

**Keywords:** avian reovirus, evolution, spillover, phylogeny, turkey reovirus

## Abstract

Avian reovirus (ARV) has emerged as an important pathogen in turkeys, causing economic losses through tenosynovitis, necrotizing hepatitis, immunosuppression, and enteric disease. Despite its ubiquity, the evolutionary history of ARV cross-species transmission among chickens, turkeys, and wild birds remains poorly understood, hindering effective control and surveillance. This study investigates ARV temporal phylogenetics with an emphasis on interspecies transmission in turkeys. Whole genome sequences (WGSs) from seventy-seven turkey cases and one quail case at the Iowa State University Veterinary Diagnostic Laboratory, along with 74–136 segment sequences per gene from GenBank (1970–2023), were analyzed. Temporal phylogenetic analyses identified chickens as the ancestral host, with spillover into turkeys beginning in the mid-20th century, followed by stable transmission within turkey populations. Migration analyses revealed predominantly unidirectional transmission from chickens to turkeys. WGS analyses showed high variability in the M2 and σC-encoding region of the S1 segment, suggesting selective pressure on outer capsid proteins. M2, S1 σC, and L3 had the highest substitution rates, implicating their role in adaptation and antigenic diversity. These findings highlight the complexity of ARV evolution across hosts and underscore the need for robust genotyping schemes and surveillance strategies to mitigate outbreaks in poultry.

## 1. Introduction

Avian reoviruses (ARVs), members of the genus *Orthoreovirus* in the family *Spinareoviridae*, are non-enveloped viruses characterized by a concentric icosahedral capsid and a segmented double-stranded RNA (dsRNA) genome [1]. ARVs have reemerged over recent decades, causing significant economic losses, primarily through tenosynovitis in meat-type poultry and necrotizing hepatitis in poults [1,2]. In addition, ARVs have been strongly associated with several other clinical syndromes, including malabsorption syndrome, runting and stunting syndrome, and immunosuppression with secondary infections [1]. However, preventing ARV-related diseases can be challenging due to the ubiquity of the virus, environmental resistance, poorly-defined pathogenesis, and the predominantly non-pathogenic nature of most field strains, which complicates virus characterization and makes it difficult to link specific strains to disease outbreaks [1,3,4]. Additionally, while horizontal and vertical transmission within domestic poultry is well recognized, the epizootiology of reoviruses in wild bird populations and their phylogenetic relationship with commercial poultry remain poorly understood. ARVs from wild birds have been found to resemble those from poultry farms, and experimental studies have shown that isolates from wild birds can cause diseases such as tenosynovitis and bursal lymphoid depletion in chickens [5,6]. This further complicates our understanding of ARV transmission dynamics and the potential role of wild birds as reservoirs or vectors for cross-species transmission.

The ARV genome is divided into 10 segments across three size classes: large (L1, L2, L3), medium (M1, M2, M3), and small (S1, S2, S3, S4) [1,7]. The large segments, L1, L2, and L3, encode the proteins λA, λB, and λC, respectively [1]. λA acts as a key scaffolding protein in the inner core shell, λB functions as the RNA-dependent RNA polymerase for transcription, and λC is positioned between the inner core and outer capsid and serves as a capping enzyme [8,9]. The medium segments M1, M2, and M3 encode μA, μB, and μNS, respectively [1]. μA is part of the inner core, μB forms the outer capsid and aids in host cell penetration, and μNS is a non-structural protein crucial for early viral morphogenesis and factory formation [8,10]. The S1 segment is tri-cistronic, encoding three proteins, σC, P10, and P17. σC, located on the outer capsid, facilitates cell attachment and contains epitopes that stimulate antibody production [1]. As the most variable ARV protein, σC significantly contributes to the challenges in developing vaccines that provide full protection to commercial flocks [11,12]. Due to this variability, σC is also widely used for ARV phylogenetic analysis and genotyping [4]. P10 is a non-structural protein with membrane-permeabilizing and fusogenic properties, while P17 is a nuclear protein with an as-yet-unknown function and might be involved with cell growth regulation [8,13,14,15]. The remaining small segments, S2, S3, and S4, encode σA, σB, and σNS proteins, respectively, with σA being an inner core protein that binds dsRNA and exhibits anti-interferon activity, σB being located on the outer capsid with an unclear function but known to be a target for neutralizing antibodies, and σNS being a single-stranded RNA-binding protein [8,16].

According to the International Committee on Taxonomy of Viruses (ICTV) species demarcation criteria, orthoreoviruses grouped into the same species should share >85% amino acid identity for conserved core proteins, >55% for the outer capsid protein, and >75% nucleotide identity for most genome segments. In contrast, viruses in different species typically exhibit <65% amino acid identity for core proteins, <35% for outer capsid proteins, and <60% nucleotide identity [17,18]. In this context, ARV isolates show significantly greater nucleotide diversity, particularly in the M2 and S1 segments, where nucleotide identity ranges from 54% to 95%, exceeding that of other orthoreoviruses (e.g., mammalian reovirus) [17,18]. In addition to genomic identity, with regard to biological properties, ARVs have been known to cause diseases in both chickens and turkeys, as well as several non-traditional poultry species and wild birds [19,20,21,22]. However, the evolutionary dynamics of ARVs across various hosts remain poorly understood, especially when compared to other segmented RNA viruses, like the influenza virus, which is well-documented to evolve in multiple hosts due to the distinct roles of its genomic segments, such as hemagglutinin [23]. In addition, similar to influenza viruses, ARVs also undergo reassortment, driving increased viral diversity, which has been increasingly reported in recent studies [24,25,26]. Surveillance at the interface between waterfowl and terrestrial birds may be valuable for monitoring emerging reoviruses. These factors underscore the complexity of ARV evolution, making epidemiological studies and risk assessments for cross-species transmission particularly challenging yet essential.

This study aims to investigate the phylogenetic evolution of ARV segments, with a particular focus on cross-species transmission and host adaptation in turkeys utilizing our database, predominantly comprising turkey-hosted ARV sequences. Understanding these spillover transmission events will aid in the development of a robust genotyping scheme and enhance surveillance and control strategies in poultry management in the future, as well as improve our understanding of viral evolution in multi-host systems.

## 2. Materials and Methods

### 2.1. Samples and Real-Time Reverse Transcription Polymerase Chain Reaction (qRT-PCR)

A total of 78 diagnostic cases submitted for ARV testing from 10 states across the US during 2019–2024, representing a wide geographic distribution, were archived at the Iowa State University Veterinary Diagnostic Laboratory (ISU-VDL) (Supplementary Table S1). These included the disease diagnostic investigations (n = 44) in which the information on tissue types (such as tendon, liver, heart, and intestines), clinical signs, or necropsy findings was available, as well as the surveillance cases (n = 34), which primarily included liver tissue. For each case, the tissue samples from the submitted flocks were collected as a pool and processed using a 2010 Geno/Grinder (SPEX, Metuchen, NJ, USA) to create homogenates for viral RNA isolation. Viral RNA was extracted from the homogenate using the MagMAX^TM^ Pathogen RNA/DNA kit (ThermoFisher Scientific, Waltham, MA, USA) following the manufacturer’s instructions. The isolated RNA was tested for ARVs by the published real-time qRT-PCR protocols, with primers and probes targeting the conserved region of the M1 genome [27].

### 2.2. Virus Isolation

Fresh samples with positive qRT-PCR results (cycle threshold (Ct) value < 36) were subjected to viral isolation following the published protocol [28]. Briefly, the fresh homogenized tissues were centrifuged at 1200 rpm for 5 min, and the supernatant was filtered through a 0.45 μm filter. The filtrate was inoculated into LMH cells (ATCC Number CRL-2117, ATCC, Manassas VA) grown in 6-well plates and incubated at 37 °C with 5% CO_2_ in a humidified incubator for 1–2 h. Subsequently, the inoculum was removed, and the fresh virus isolation medium was added. The plates were incubated at 37 °C with 5% CO_2_. The cells were checked for the appearance of cytopathic effect (CPE) over a period of seven days. When ~70–80% of viral CPEs were observed, the plates were subject to two freeze–thaw cycles before harvesting. If no CPE was observed, the plates were subject to two freeze–thaw cycles before harvesting. The cell lysates were centrifuged at 2000 rpm for 10 min, and the supernatants were harvested. The supernatants were tested by ARV real-time RT-PCR, as described above. The positive virus isolates were passaged once before being stored at −80 °C.

### 2.3. Whole Genome Sequencing (WGS)

The viral RNA was extracted using the MagMAX^TM^ pathogen RNA/DNA kit (ThermoFisher Scientific, Waltham, MA, USA) with the KingFisher Flex system. Double-stranded cDNA was synthesized using the NEXTflex^TM^ Rapid RNA-Seq Kit (Bioo Scientific Corp, Austin, TX, USA) [29]. The sequencing library was prepared using the Nextera XT DNA library preparation kit (Illumina, San Diego, CA, USA) with dual indexing. The pooled libraries were sequenced on an Illumina MiSeq platform at the next-generation sequencing (NGS) section at ISU-VDL with the 600-Cycle v3 Reagent Kit (Illumina) to generate 300 base paired-end reads by following standard Illumina protocols. The raw reads per sample were quality checked with FastQC (v0.11.9) and processed with Trimmomatic (v0.39) [30]. High-quality reads were aligned to reference sequences for each segment of avian reovirus obtained from the National Center for Biotechnology Information (NCBI) database using BWA-MEM (v 0.7.17) [31]. The reads mapped to the reference sequences were extracted using SAMtools (v1.7) [32] and de novo assembled using ABySS (v2.2.4) [33] and SPAdes (v3.13.0) [34]. The final assemblies were checked using BLASTn (NCBI; https://blast.ncbi.nlm.nih.gov/blast/Blast.cgi) and manually curated in IGV (v2.16.0) to obtain a consensus sequence for each segment [35].

### 2.4. Data Acquisition and Curation

ARV sequences that represented >90% of the full-length open reading frame (ORF) of each segment were selected, and the alignments were carried out using MAFFT v7.490 in Geneious Prime 2023 (Biomatters) [36]. Maximum likelihood (ML) phylogenetic trees (IQ-TREE) were generated for each segment using IQ-TREE v2.3.6 using a general time reversible model [37], and tree topologies were validated with 1000 bootstrap replicates. Metadata such as host species, location, lab accession number, tissue of isolation, and time of sampling were collected when available, and the nomenclature of the isolate was based on the consensus designed as species/geographic region/lab identification number/tissue of isolation/year of isolation [38]. For temporal evolution and spillover analysis, a subset of sequences representing each clade of each segment was selected ad hoc and queried against the NCBI nucleotide BLAST database, with a maximum of 100 target sequences per query. This initially yielded 700–1200 sequences, depending on genome segments. After removing duplicates and sequences with more than 10% missing nucleotides in the full-length ORF, 74 to 136 sequences from GenBank were retained. Sequences and paired metadata, such as host species and sampling time, were also retrieved from GenBank.

### 2.5. Phylogenetics and Temporal Reconstruction of ARV Transmission

The ORFs of each ARV segment from ISU-VDL, combined with 74 to 136 sequences obtained from GenBank spanning the years 1970 to 2023, were used to generate a phylogenetic ML tree (IQ-TREE). Temporal signals, estimation of the evolutionary rate, and origin time for the ML phylogenies were investigated using a linear regression of root-to-tip genetic distances against sampling dates in TempEst v1.5.3 [39] and ML dating in TreeTime [40]. Timed phylogenies of the ML trees of each segment were inferred using TreeTime. In addition, to understand the transmission pattern between different host species, TreeTime “mugration” analysis was performed by treating hosts as discrete characters. The transitions were modeled as a time-reversible process, with comparable sampling probabilities for the different host species. The models infer migration dynamics between hosts, with the symmetrized migration rate reflecting the strength of bidirectional transmission, while the actual migration rate indicates the weight of unidirectional transmission. The phylogenies were visualized using FigTree v1.4.4 [41].

### 2.6. Reassortment, Recombination, and Selective Pressure Analyses

Reassortment of M genes was screened using TreeSort [42]. Recombination events of the complete M2 and σC were assessed by the Genetic Algorithm for Recombination Detection (GARD) through the Datamonkey online service [43]. Selective pressure on the complete M2 and σC datasets was evaluated using a combination of evolutionary analyses methods, including the Mixed Effects Model of Evolution (MEME) [44], the Fixed Effects Likelihood (FEL) [45], and Fast Unconstrained Bayesian AppRoximation (FUBAR) [46], which were all implemented through the Datamonkey online service [47]. These algorithms estimate diversifying or purifying selection with significance thresholds set at *p* < 0.1 for the MEME and FEL and 0.9 for FUBAR based on the ratios between non-synonymous substitution rates (dN, beta) and synonymous substitution rates (dS, alpha). As the results can differ significantly depending on the chosen method, we only considered sites that were under positive selection pressure according to all three of the aforementioned methods.

### 2.7. Statistics

qRT-PCR Ct values were compared between diagnostic sample types (liver and tendon), as well as between liver samples with different submission purposes (surveillance vs. diagnostic), using Welch’s *t*-test. Data are presented as mean ± standard deviation in box and whisker plots. All statistical analyses were performed using GraphPad Prism 9 (GraphPad Software Inc., San Diego, CA, USA), with *p* < 0.05 considered statistically significant.

## 3. Results

### 3.1. Clinical Data

The common tissue types for diagnostic investigation cases involving WGS and/or virus isolation were from the liver (n = 23) and tendon (n = 13) and were frequently associated with lesions of necrotizing hepatitis or lymphoplasmacytic/lymphohistiocytic tenosynovitis, and Ct values in liver samples (mean 16.9 ± 4.5, range 11.2 to 27) were significantly lower than tendon samples (mean 25.1 ± 2.9, range 19.1 to 29) (*p* < 0.0001) (Supplementary Figure S1). For surveillance samples, the liver was the most commonly received sample type (n = 18), followed by the heart (n = 5) and tendon (n = 5). The surveillance liver samples had a mean Ct value of 20.2 ± 6.6 (range 9.5 to 35.7), which was higher than the diagnostic samples (16.9 ± 4.5) (*p* = 0.057).

Among the 78 samples with WGS determined in this study, the majority (66/78) were cell culture isolates. The positive virus isolation results were confirmed by the presence of cytopathic effects (syncytia) in inoculated cell culture and the positive qPCR results on the virus isolates with Ct values ranging from 12.5 to 22.3. The other 12 sequences (12/78) were determined directly from the clinical samples with Ct values of 13.2 to 26.8 (Supplementary Table S1).

### 3.2. ARV Nucleotide Homology

In comparative genome analysis of the 78 cases submitted to ISU-VDL, a subset of seven cases involved multiple organ submissions for WGS, with two to four organs per case. To ensure data integrity, a quality control measure was implemented and excluded segments with more than 10% missing nucleotides in the full-length ORF. As a result, up to five segments per case were eliminated. Additionally, more than one sequence was identified for some segment(s) in some cases, suggesting the presence of co-circulating ARVs in the tested flocks (Table 1; Supplementary Table S1).

A final total number of 88 to 93 sequences from the ISU-VDL database was included for analysis and revealed significant genetic heterogeneity among ARVs isolated from the US (Table 1). The overall nucleotide homology values of the sequences from the ISU-VDL database were higher than 90.6% for L1, 93.2% for L2, 91.4% for L3, 90.1% for M1, 64.0% for M2, 86.0% for M3, 42.6% for the σC-encoding region of S1, 90.1% for S2, 84.7% for S3, and 88.6% for S4 segments (Table 1). After quality confirmation on sequences from GenBank, a total of 74 to 136 sequences from GenBank were compared with the L1 to S4 segment sequences from the ISU-VDL database. Nucleotide distance analysis revealed that sequences from the ISU database exhibited a wide range of nucleotide homology with sequences reported globally (Table 1). 

### 3.3. Phylogeny of ARV Segments and Spillover Investigation

#### 3.3.1. Chicken as a Common Ancestral Host for ARVs in Turkeys

**Figure 1:**
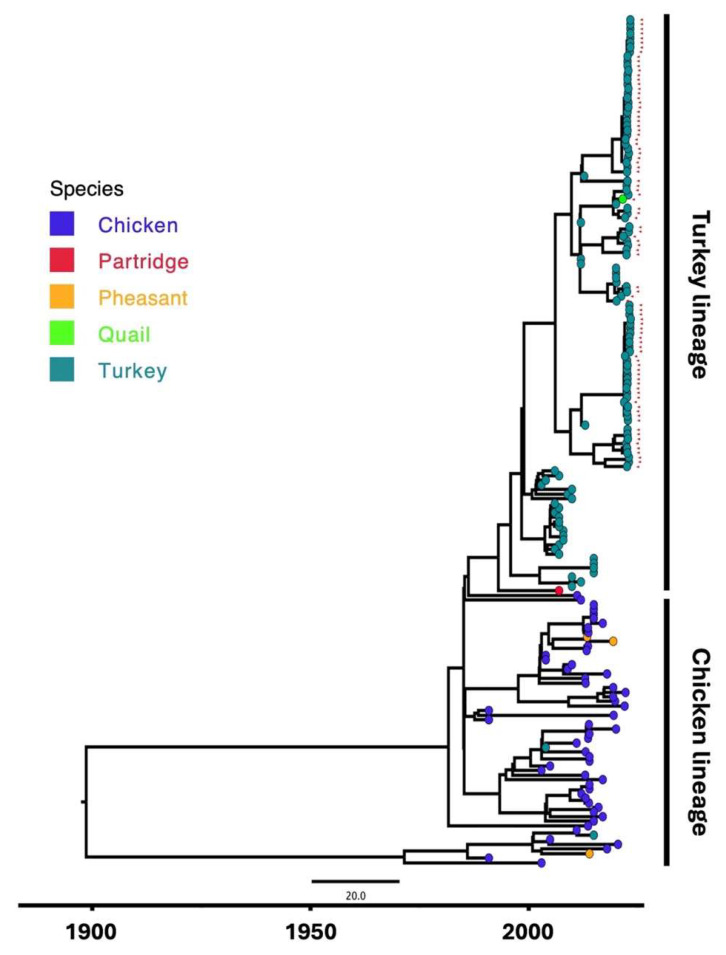
Time-calibrated maximum likelihood phylogenetic tree reconstructed using the L3 sequences of all strains included in the study. The host species are color-coded. Diversification into two major lineages (chickens and turkeys) is seen across ten segments, except M2 and the S1 σC-encoding region. Self-sampled sequences are indicated by red arrowheads.

Phylogenetic analysis identified an evolutionary history that was found to be similar across ten of twelve segments, except M2 and the S1 σC-encoding region. These eight segments share a similar number of cross-species events in history as well as the involved host avian species (Figure 1 and Supplementary Figure S2). Taking L3 for example, phylogeny suggests that the time to the most recent common ancestor (tMRCA) dates to the late 1800s (Figure 1; Table 2). From this root, initial diversification occurred in the early 1900s with major divergences that gave rise to two prominent lineages: one associated with chicken as the predominant host and another comprising a mix of turkey– and chicken–host viruses. This pattern of the phylogeny with two major divergences corresponding to the two main avian hosts (chicken and turkey) was observed consistently across L1, L2, M3, S2, and S4 segments. A similar but fairly distinct pattern is seen in the M1 and S3 segments, where the initial divergence from the root directly gives rise to two major lineages corresponding to the two primary hosts (chickens and turkeys) (Supplementary Figure S2).

#### 3.3.2. M2 Has a Unique Evolutionary History

**Figure 2:**
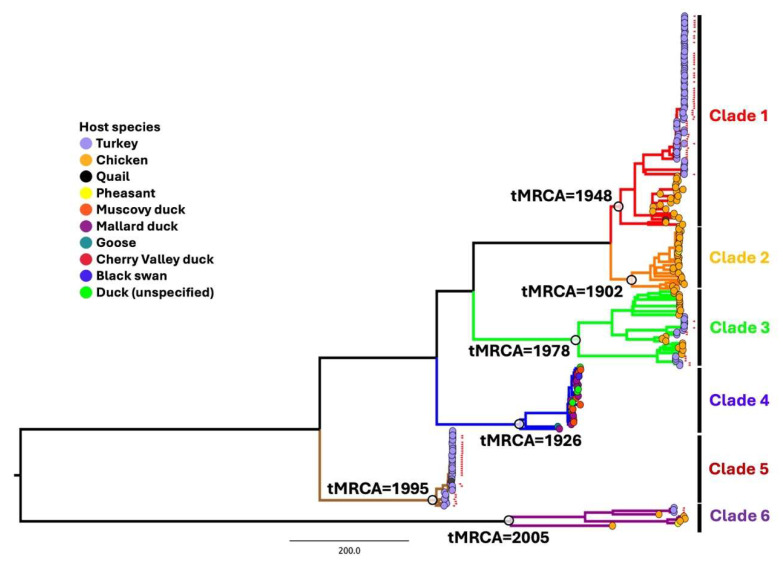
Maximum likelihood phylogenetic tree of the M2 segment. The tree reveals diversification into six major clades, with the time of the most recent common ancestor (tMRCA) indicated at the roots of each clade. Clades 2 and 5 are predominantly associated with chickens and turkeys, respectively, while clade 4 consists exclusively of waterfowl species. Clades 1, 3, and 6 include a mix of both chicken and turkey hosts. Host species and clades are color-coded. Self-sampled sequences are indicated by red arrowheads.

The M2 segment exhibits distinctly different clades with a wide range of avian species involved and a considerable root-to-tip distance variation (R^2^ = 0.06) revealed by both TempEst and TreeTime analyses, suggesting different virus mutation rates of M2 across virus populations. To address this, residue distribution was analyzed and found to correspond to six major clades (Supplementary Table S2). Subsequently, TempEst and TreeTime analyses were performed separately for each clade. Clades 2 and 5 of the M2 segments are predominantly identified in chicken and turkey hosts, respectively (Figure 2). Clade 2 appears to have a longer evolutionary history, with its tMRCA tracing back to the early 1900s in chicken hosts, and it is presumed to represent the ancestral origin of M2 (Table 2). Clade 4 hosts are exclusively waterfowl, such as Muscovy ducks and mallard ducks. Clades 1, 3, and 6 include a mix of both chicken and turkey hosts, with chickens estimated to be their ancestor host for these three clades. Clade 5 and clade 6 are found to occur more recently, with the root converging back to 1995 and 2005, respectively. Comparative sequence analysis between these six clades ranges from approximately 65% to 90% nucleotide similarity. Clade 6 is notably more genetically and phylogenetically distinct and shares 32% to 34% nucleotide dissimilarity with other clades.

#### 3.3.3. The S1 σC-Encoding Region in Turkeys Is Estimated to Have Differing Common Ancestors Compared to Other Segments

**Figure 3:**
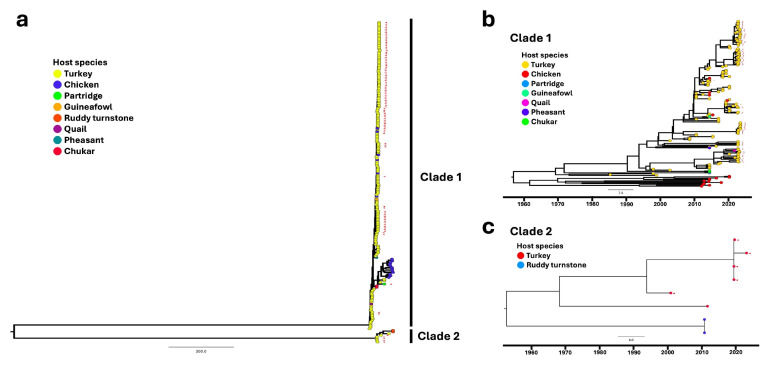
Maximum likelihood phylogenetic tree (**a**) of the S1 σC-encoding region. The two major clades are composed primarily of turkey hosts with a few chickens and wild birds (**b**) and composed of turkey hosts and ruddy turnstones (**c**), respectively. Host species are color-coded. Self-sampled sequences are indicated by red arrowheads.

In this study, we identified two major clades with significant genetic differences (Figure 3a; Supplementary Figure S3): the larger clade (clade 1, Figure 3b; Supplementary Figure S3b), predominantly composed of sequences from turkey hosts with a few chicken hosts, and the smaller clade (clade 2, Figure 3c; Supplementary Figure S3c), comprising turkey and ruddy turnstone hosts. For both clades, in contrast to the early 1900s for other segments, the tMRCA of the σC segment is estimated to have occurred later, around the 1950s. In clade 1, the initial diversification events for the segment were estimated to have occurred in the late 1950s and 1960s, marking the beginning of distinct evolutionary trajectories within poultry. Notably, the root of the chicken lineage is older than that of the turkey lineage, while the oldest isolates (leaves) from turkey hosts precede those from chickens. This suggests that the ancestral turkey S1 likely originated from the chicken source that was unobserved.

After the initial spillover, lineages diverged, and the stable transmission of the segment within the turkey host was established, as observed with most other segments from the 1990s to the 2010s. Notably, in the 2010s, multiple sporadic S1 segments of chicken origin were found within the turkey lineage, suggesting transmission of this segment between chicken and turkey (Figure 3b).

In this study, we identified a genetically and phylogenetically distinct group of strains (clade 2) isolated from turkeys (n = 6) that are more closely related to S1 from ruddy turnstones and share only approximately 45% nucleotide similarity with other ARVs. The tMRCA for this group also dates back to the 1950s. ARVs from ruddy turnstones have been reported to be closely related to Tvärminne avian virus, a novel Orthoreovirus identified in Finland in a crow with neurological signs [48,49]. In addition, one of the isolates in this group (NC/SEP-R44/03) was previously proven to be highly pathogenic in turkeys through an animal challenge study [50]. However, all ARV isolates from ISU-VDL in this study were surveillance samples (intestine and liver) with unknown disease status.

#### 3.3.4. Estimated Higher Nucleotide Mutation Rates in L3, M2, and the S1 σC-Encoding Region

Nucleotide mutation rates analyzed by TreeTime and TempEst for the ten segments were comparable and summarized in Table 2. While most of the virus mutation rates are similar, L3, M2 clade 6, and the S1 σC-encoding region appear to have higher mutation rates in this analysis. M2 clade 6 has the highest estimated mutation rate (TreeTime, 2.07 × 10^−2^ nucleotide substitutions/site/year, n/s/y; TempEst, 2.33 × 10^−2^ n/s/y), followed by the L3 segment (TreeTime, 5.21 × 10^−3^ n/s/y; TempEst, 4.624 × 10^−3^ n/s/y) and the S1 σC-encoding region (TreeTime, 2.45 × 10^−3^ n/s/y; TempEst, 2.49 × 10^−3^ n/s/y).

#### 3.3.5. Cross-Species Transmission Predominantly from Chickens to Turkeys and Other Avian Species

**Figure 4:**
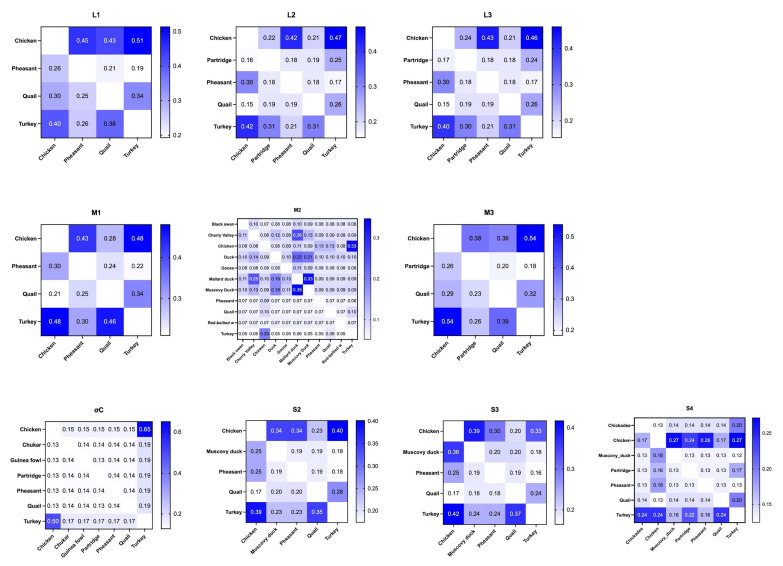
Heatmap of actual migration rates between different host species (from x- to y-axis) across ten ARV segments. The numbers in the blocks represent the calculated migration rate between hosts. Transmission from turkeys to chickens is more pronounced in the L2, L3, M2, S1 σC-encoding region and the S2 and S4 segments, while it is nearly equal in the M1 and M3 segments. Additionally, transmission from turkeys to chickens is observed in the S3 segment.

The segment transmission between hosts and transmission rates of these sequences were analyzed by TreeTime “mugration” analysis. The actual migration rates (asymmetric migration rate), which reflect the weight of unidirectional transmission, indicate transmission directions. There appears to be a directional trend in virus segment transmission between different host species, though it is not particularly pronounced (Figure 4). Using L1 as an example, a higher portion of transmission occurs from chickens to turkeys (actual rate = 0.51) and other avian species, such as pheasants (0.45) and quail (0.42), while a smaller portion flows from turkeys to chickens (0.40). This transmission pattern from chickens to turkeys was consistently observed in segments L2, L3, M2, S1, S2, and S4. Conversely, segments M1 and M3 exhibited nearly equal transmission rates between chickens and turkeys (transmission rate difference < 0.002), suggesting no significant directional bias. Notably, the S3 segment displayed a higher migration rate from turkeys to chickens (0.42) compared to chickens to turkeys (0.33), highlighting a unique transmission dynamics pattern in this segment.

#### 3.3.6. Positive Selective Pressure on σC Protein

Given the observed lowest nucleotide homologies in the S1 σC-encoding region and M2 genes, and to understand the evolutionary forces shaping these genes and their potential implications for viral fitness and host interactions, we investigated the recombination and selective pressures acting on σC and M2. Sequences were screened for recombination using GARD, which detected no evidence of breakpoints. Analysis of selective pressure on the σC protein identified significant diversifying selection at twenty-seven codons via MEME, four codons via FEL, and three codons via FUBAR (Table 3; Supplementary Table S3). Notably, there was agreement across all methods for diversifying selection at codons 117, 148, and 273. Residues 117 and 148 are within the alpha-helical coiled coil domain, while residue 273 is located on the outward-facing surface of the beta-barrel carboxy-terminal globular head. Selective pressure analysis of the M2 (μB) protein revealed diversifying selection at 46 codons, as identified by the MEME, whereas no codons were identified under diversifying selection by FEL or FUBAR (Supplementary Table S3).

## 4. Discussion

Avian reovirus has caused significant economic losses in the poultry industry, highlighting the urgent need to clarify current transmission patterns through molecular epidemiology across different hosts, particularly given the limited data currently available for turkey hosts. To address this, we reconstructed the temporal phylogeny of ARVs by genomic segments, investigated interspecies transmissions, and estimated the substitution rates for each segment. Our findings indicate that chickens are the ancestral host with spillovers into turkeys, followed by stable transmission within the turkey population. Moreover, in this study, we characterized and identified the M2, σC, and L3 genes as exhibiting higher genetic variability and the highest substitution rates. These findings would provide foundational data for the development of a robust genotyping scheme to inform future disease prevention strategies.

In this study, we first found that the diagnostic samples exhibited lower Ct values compared to surveillance samples, though the difference was not statistically significant. This suggests that diagnostic samples were more likely to be associated with lesions potentially linked to viral infection, as they originate from cases where clinical disease was suspected. Currently, no genetic markers have been identified to differentiate pathogenic from non-pathogenic ARVs [4], and it is well known that the majority of ARVs are considered non-pathogenic [3]; therefore, information on whether viruses isolated from these active surveillance samples are associated with clinical disease could not be determined. Although the significance of virus isolates from surveillance samples remains unclear, they still contribute valuable information to the growing database for epidemiology research and lead to a future research direction involving the genomic comparison between isolates from lesions and non-lesions to identify potential pathogenicity markers. This could aid in diagnosing and distinguishing between pathogenic and non-pathogenic strains, thereby further contributing to the selection of future vaccine candidates.

ARV nucleotide homology analysis revealed that the M2 and S1 σC-encoding regions displayed the greatest genetic variability. These findings align with previous phylogenetic analyses of ARVs, which indicated that S1 and M2 as the most genetically diverse segments [4,8,43,45,46,47]. More specifically, M2 encodes the major outer capsid protein μB, which forms the outermost layer of the virion, while σC serves as the viral cell attachment protein [8,43,44]. These virus surface proteins play key roles in capsid stability, host interaction, and immune response, factors likely driving their higher mutation rates and experiencing greater selective pressure [12]. Therefore, to investigate the selective pressures acting on these two segments, we conducted a selective pressure analysis and identified evidence of positive selection within a B-cell epitope of the σC protein. This finding highlights the role of selective pressure in driving diversification at antibody-binding sites, potentially enabling immune evasion through increased sequence variability [12,51]. Previous studies show that different ARV genotypes in chicken isolates exhibit higher episodic diversification in both stalk regions and globular head domains [25,52]; however, the affected residues in our study differ from those reported, suggesting different evolutionary strategies of ARVs adopted to interact with different hosts or immune environments. The M1 and S2 segments were the most genetically conserved. M1 encodes μA, a putative transcriptase co-factor, while S2 encodes σA, a protein responsible for dsRNA binding and anti-interferon activity [8]. These conserved viral genes are associated with genome regions critical for the virus lifecycle, such as replication, packaging, and maintenance of genetic or virion structure [48]. The essential roles of μA and σA in transcriptional support likely contribute to this observation, with evolutionary pressures maintaining their functions and reducing genetic diversity.

In the time-scaled phylogenetic analysis of all ten segments, except M2 and σC, turkey-hosted ARVs shared a common ancestor with historical chicken-hosted ARVs in a time-scaled phylogenetic tree. This suggests that the turkey-hosted lineage likely originated from an initial spillover event from the chicken-hosted lineage, with the estimated timing varying by segment, ranging from the 1940s to the 1980s, depending on the segment. Following the initial spillover, ARV segments in turkeys appear to have formed distinct lineages within the turkey host, indicative of stable transmission chains within the turkey host and subsequent diversification. This study also found a higher estimated effective population size by coalescent models in the time-scaled phylogenetic tree, along with more divergence observed in the ML tree (Supplementary Figure S4). This could reflect the high diversity of the virus population, possibly due to outbreaks, new introductions of virus from other avian species, reassortment events, and/or suggest an increase in sampling or enhanced monitoring of these viruses in commercial poultry [25,26,53]. Although skyline population curves cannot be considered a strict proxy for viral prevalence, the viral population dynamics inferred through coalescent models, combined with temporal trends from time-scaled phylogeny, offer insights into the evolutionary history of the virus.

In cases of cross-species transmission, this initial spillover event marks a critical step in the emergence of an epidemic or endemic. Successful infection of a new host requires acquiring key mutations to overcome barriers such as host entry receptor compatibility, adaptation to proteases and protein modifications, immune evasion, optimized transmission dynamics, and population-level stability [54]. Although the findings of this study suggest that turkey ARV isolates predominantly diverged from chicken-derived sequences, consistent with prior research [55], this does not conclusively establish whether any ARV isolates can readily infect different poultry species or other avian hosts. Experimental studies have yielded variable results, showing that antibody responses may or may not develop in turkeys inoculated with viruses from chickens, and vice versa [11,56].

For the phylogenetic analysis in this study, it should be noted that the inference of virus evolution is segment-based and relies heavily on data from GenBank, anchored by our dataset and predominantly composed of ARV sequences from turkey hosts. Also, to better illustrate the phylogeny of ARVs in turkey hosts, efforts were made to include as many sequences as possible from the GenBank database that are particularly genetically related to the turkey-hosted ARVs in our dataset. As a result, no strict nucleotide similarity cutoff was established regarding the number of sequences or host species to be included for phylogenetic evaluation. Therefore, despite thorough data collection methods, this approach might introduce potential preanalytical bias, leading to an overrepresentation of turkey-derived sequences and an underrepresentation of those from chickens. Additionally, not all avian-hosted ARV strains or genome segments, especially those genetically distant from turkey-host ARVs, were included. Nonetheless, this approach may provide a more accurate reflection of ARV evolution, particularly in turkey-hosted strains, by ensuring sufficient quality while accounting for genetic variation.

The M2 tree topology, in which chicken and turkey lineages are mixed (especially in clades 3 and 6), is distinct from other segments, where the virus forms separate lineages for each host (chicken vs. turkey). This is possibly due to reassortment events between different strains from different hosts, particularly between chicken and turkey reoviruses [25,26,57]. Initial screening of the M1, M2, and M3 genes using TreeSort did not identify any cross-species reassortment events. The presence of mixed chicken and turkey lineages may also be due to the limited sample size available from different host species. Interestingly, reassortment events have not been detected between galliform and waterfowl hosts, suggesting that the M2 segment exhibits host-specific evolutionary patterns within these distinct host groups (galliforms vs. waterfowl) [26,57]. We also found that M2 clade 6, including several isolates from turkey hosts at ISU-VDL, is the most phylogenetically distinct from other clades. Strains in clade 6 were initially detected in chickens in Hungary from 2005 to 2011 and also shown as a monophyletic group distinct from waterfowl and other gallinaceous ARVs in the previous report [26]. This distinction suggests that these strains may have acquired this gene through reassortments, possibly involving different geographic locations and an unknown host species, followed by an adaptation process to galliform hosts, specifically chickens in Hungary and turkeys in the United States [26].

For the σC phylogeny, in addition to ruddy turnstones, there have been scattered spillovers from poultry to non-poultry hosts, including quails, pheasants, partridges, chukars, and guineafowls. The hypothesis that wild birds contribute to the maintenance and transmission of avian reoviruses has been previously proposed based on findings of genetic links between strains in wild birds and those affecting commercial poultry, as well as sporadic similar clinical signs identified as seen in commercial poultry species [6]. In the affected non-poultry birds, intestinal disturbances have been reported in partridges [20] and pheasants [58], while quails may exhibit enteritis and hepatitis [21,59]. Additionally, splenitis, hepatitis, myocarditis, arthritis, and tenosynovitis have been reported in waterfowl [22]. In this study, based on the phylogeny and cross-species transmission analysis, reoviruses in these non-poultry avian species appear to be acquired from poultry and maintained within these species due to limited evidence of spillover back to poultry or associated outbreaks. However, these avian species may still act as reservoirs and potentially facilitate further virus transmission, and these dynamics also highlight the complex interactions between different host species in the transmission cycle and require further investigations. In addition, for the S1 phylogenetic analysis, it is important to note that traditional Kant’s genotyping was not employed in this study, as the focus was on investigating evolutionary history based on the genetic relatedness of ARVs using turkeys as the primary host and anchor for comparison. In addition, Kant’s σC genotyping method mainly groups turkey reoviruses into genotype Cluster 2, failing to provide higher resolution for their genetic characterization [28,55,60]. Therefore, a more refined typing method, particularly for turkeys, is needed to allow for better differentiation and a deeper understanding of turkey reovirus evolution.

This study is the first attempt to estimate molecular evolution rates across all ten segments of ARVs, all of which fall within the estimated evolutionary rates for RNA viruses. For instance, typical rates in Rotavirus and influenza virus are over 1 × 10^−3^ n/s/y, compared to low mutation rates in Flavivirus, for example, at 1 × 10^−6^ [61,62]. We found that the nucleotide mutation rates estimated by TreeTime and TempEst were comparable across all ten segments (Table 2), although slight differences may exist between the TreeTime and TempEst methods. While both are based on root-to-tip regression, TreeTime is considered more comprehensive, as it utilizes a maximum likelihood-based regression algorithm, whereas TempEst employs an iterative regression approach [39,40]. That said, we found that the temporal signals in this study, as well as in previously published reports, were not exceptionally strong. Additionally, we observed discrepancies in tMRCA estimates (Table 2). This finding may be attributed to factors such as inaccurate metadata submitted to GenBank, a lack of data from a long enough time span (short evolutionary timescale), and the occurrence of multiple segment-based evolutionary events, including reassortment and purifying or positive selective pressures [24,25,53,63]. Additionally, one consideration in the nucleotide mutation rate analysis of M2 is that in order to achieve a better temporal signal, the sequences were subgrouped, resulting in a low number of sequences analyzed in some clades. This low sample size may have also contributed to a higher mutation rate observed in M2 clade 6. Nevertheless, the positive correlation between genetic divergence and sampling time observed in all segments indicates their suitability for phylogenetic analysis [39]. With the information on phylogeny and nucleotide substitution rates, insights into virus evolution are still enhanced, as this information could provide better predictions of nucleotide changes and circulating strains, and, therefore, could further inform surveillance efforts and the development of targeted interventions [64].

## 5. Conclusions

This study provides the evolutionary history of ARVs with a focus on cross-species transmission, particularly in turkeys. To date, this is the most comprehensive reconstruction of the evolution of turkey ARVs using all gene segments from available data. Phylogenetic analyses identified chickens as the ancestral host of ARVs in turkeys; however, understanding the mechanisms of viral transmission across hosts remains challenging due to the complexity that arises from factors such as vaccine usage and protective efficacy, horizontal and vertical integration of farming systems, interactions between commercial poultry species and wild birds within multi-host ecology of ARVs, and the virus’ sophisticated evolutionary strategies. The historical depth of turkey ARV segments available for analysis was a confounding factor in the analysis, which prospective sequencing efforts could improve. To prevent disease transmission, efforts should prioritize identifying genomic markers of pathogenicity, improving epidemiological monitoring, and investigating ecological and human-driven factors influencing transmission events. These strategies will enhance surveillance, optimize vaccine efficacy, and mitigate the economic impact of ARVs in poultry production systems.

## Figures and Tables

**Table 1:** Avian reovirus nucleotide homology.

Data Source		L1	L2	L3	M1	M2	M3	S1 ^a^	S2	S3	S4
ISU-VDL	Total sequence number in the analysis	90	88	88	90	92	92	92	89	93	89
	Number of co-detection sequences	5	5	6	5	6	9	5	5	6	4
	Additional sequences from the same case accession in different organ(s)	9	9	9	9	9	9	9	9	9	9
	Sequences with >10% missing nucleotides in full-length ORF	2	4	5	2	1	4	1	3	0	2
	Nucleotide similarity (%)	90.6–100	93.2–100	91.4–100	90.1–100	64–100	86–100	42.6–100	90.1–100	84.7–100	88.6–100
	Amino acid similarity (%)	94–100	93.0–100	92.7–100	93.1–100	65.3–100	91.5–100	31.8–100	96.7–100	90.6–100	94.0–100
GenBank	Total sequence number in the analysis	108	107	96	103	136	108	102	99	74	95
All	Total sequence number	198	195	184	193	228	200	194	188	167	184
	Nucleotide similarity (%)	81.8–100	80.6–100	71.0–100	83.7–100	61.8–100	77.6–100	42.1–100	85.2–100	67.2–100	76.4–100
	Amino acid similarity (%)	92.3–100	88.8–100	78.0–100	92.0–100	63.04–100	86.0–100	29.0–100	89.2–100	72.8–100	80.6–100
	Host species	Chicken, Pheasant, Turkey, Quail	Chicken, Pheasant, Turkey, Quail, Partridge	Chicken, Pheasant, Turkey, Quail, Partridge	Chicken, Pheasant, Turkey, Quail	Turkey, Chicken, Quail, Pheasant, Muscovy Duck, Mallard Duck, Goose, Cherry Valley Duck, Black Swan, Duck (Unspecified)	Chicken, Pheasant, Turkey, Quail	Turkey, Chicken, Partridge, Guineafowl, Ruddy Turnstone, Quail, Pheasant, Chukar	Chicken, Pheasant, Turkey, Quail, Muscovy Duck	Chicken, Pheasant, Turkey, Quail, Muscovy Duck	Chicken, Pheasant, Turkey, Quail, Muscovy Duck, Chickadee, Partridge

^a^ σC-encoding region.

**Table 2:** Estimated time of most common recent ancestors (tMCRAs) and nucleotide mutation rates (substitutions/site/year, n/s/y) of each avian reovirus segment.

TreeTime				TempEst			
	tMCRA	Mutation Rate (n/y/s)	R^2^		tMCRA	Mutation Rate (n/y/s)	R^2^
L1	1890	1.62 × 10^−3^	0.62	L1	1896	1.74 × 10^−3^	0.48
L2	1883	1.37 × 10^−3^	0.32	L2	1918	1.91 × 10^−3^	0.39
L3	1895	4.62 × 10^−3^	0.29	L3	1901	5.21 × 10^−3^	0.32
M1	1941	1.42 × 10^−3^	0.49	M1	1948	1.90 × 10^−3^	0.48
M2-1 ^a^	1948	1.18 × 10^−3^	0.6	M2-1	1947	1.91 × 10^−3^	0.61
M2-2 ^b^	1902	9.61 × 10^−4^	0.35	M2-2	1910	1.03 × 10^−3^	0.19
M2-3 ^c^	1978	1.80 × 10^−3^	0.41	M2-3	1968	8.19 × 10^−3^	0.3
M2-4 ^d^	1926	9.03 × 10^−3^	0.51	M2-4	1912	9.27 × 10^−4^	0.51
M2-5 ^e^	1995	1.53 × 10^−3^	0.95	M2-5	2001	2.14 × 10^−3^	0.92
M2-6 ^f^	2005	2.07 × 10^−2^	0.65	M2-6	2006	2.33 × 10^−2^	0.7
M3	1834	1.24 × 10^−3^	0.41	M3	1834	1.24 × 10^−3^	0.41
S1-1 ^g^	1952	2.45 × 10^−3^	0.56	S1-1 ^a^	1952	2.50 × 10^−3^	0.56
S1-2 ^h^	1949	1.48 × 10^−3^	1	S1-2 ^a^	1951	1.58 × 10^−3^	0.83
S2	1920	1.36 × 10^−3^	0.44	S2	1920	1.36 × 10^−3^	0.44
S3	1698	1.63 × 10^−3^	0.4	S3	1683	1.56 × 10^−3^	0.36
S4	1779	1.17 × 10^−3^	0.21	S4	1779	1.17 × 10^−3^	0.21

Clades were designated for M2 and the σC-encoding region based on residue distribution with adequate R^2^ values. Abbreviations: ^a^ M2-1, M2 clade 1; ^b^ M2-2, M2 clade 2; ^c^ M2-3, M2 clade 3; ^d^ M2-4, M2 clade 4; ^e^ M2-5, M2 clade 5; ^f^ M2-6, M2 clade 6; ^g^ S1-1, S1 σC-encoding region clade 1; ^h^ S1-2, S1 σC-encoding region clade 2.

**Table 3:** Selective pressure analysis on the σC gene using the Mixed Effects Model of Evolution (MEME), Fixed Effects Likelihood (FEL), and Fast, Unconstrained Bayesian AppRoximation (FUBAR) methods. In gray, the positions that were found to be under positive selective pressure by all methods are highlighted.

Codon	MEME			FEL			FUBAR	
	LRT	*p*-Value	Class	LRT	*p*-Value	Class	Beta/Alpha	Post. Probability
22	3.472	0.083	Diversifying	2.211	0.137	Neutral	−0.018	0.534
26	4.1	0.06	Diversifying	0.119	0.7301	Neutral	−0.011	0.477
31	8.568	0.006	Diversifying	0.014	0.9067	Neutral	−0.441	0.14
60	3.575	0.079	Diversifying	0.636	0.4251	Neutral	−0.482	0.282
79	3.695	0.074	Diversifying	0.868	0.3516	Neutral	0.129	0.614
117	5.528	0.029	Diversifying	4.683	0.0305	Diversifying	1.092	0.918
135	3.873	0.068	Diversifying	0.014	0.9056	Neutral	−0.36	0.264
148	8.224	0.007	Diversifying	2.963	0.0852	Diversifying	1.218	0.921
215	18.462	0	Diversifying	0.428	0.5131	Neutral	−0.161	0.271
240	3.132	0.1	Diversifying	12.039	0.0005	Purifying	−2.393	0
273	10.113	0.003	Diversifying	7.19	0.0073	Diversifying	1.735	0.981
281	16.147	0	Diversifying	1.662	0.1974	Neutral	−1.695	0.006
282	9.83	0.003	Diversifying	0.412	0.5211	Neutral	−1.59	0.024
289	3.603	0.078	Diversifying	0.268	0.6047	Neutral	0.13	0.613
296	8.084	0.008	Diversifying	10.615	0.0011	Purifying	−2.837	0
306	5.299	0.032	Diversifying	0.656	0.4178	Neutral	−0.637	0.092
307	4.151	0.058	Diversifying	0.411	0.5215	Neutral	−1.762	0.014
310	3.679	0.075	Diversifying	3.79	0.0516	Purifying	−2.066	0.002
316	10.144	0.003	Diversifying	0.389	0.533	Neutral	−1.676	0.025
318	10.688	0.002	Diversifying	0.993	0.319	Neutral	−1.771	0.02
319	17.884	0	Diversifying	0.59	0.4423	Neutral	−0.285	0.35
320	17.92	0	Diversifying	0.003	0.9531	Neutral	−1.315	0.078
321	7.18	0.012	Diversifying	1.276	0.2586	Neutral	0.079	0.536
325	31.05	0	Diversifying	0.076	0.7823	Neutral	−1.5	0.044
326	27.676	0	Diversifying	0.299	0.5845	Neutral	−0.697	0.197
327	8.414	0.007	Diversifying	5.275	0.0216	Diversifying	0.236	0.725
328	25.523	0	Diversifying	0.974	0.3237	Neutral	−0.236	0.313

Significance values are set at 0.1 for the MEME and FEL and 0.9 for FUBAR. LRT, likelihood ratio test statistic for beta = alpha (neutral hypothesis) vs. beta ≠ alpha (alternative hypothesis). Beta, non-synonymous substitution rate. Alpha, synonymous substitution rate.

## Data Availability

Viral genome sequences were deposited in the NCBI GenBank (https://www.ncbi.nlm.nih.gov/genbank/) under accession numbers PV405945–PV406034 (L1 gene), PV406035–PV406122 (L2 gene), PV406123–PV406210 (L3 gene), PV406211–PV406300 (M1 gene), PV406301–PV406392 (M2 gene), PV406393–PV406484 (M3 gene), PV406485–PV406575 (S1 gene), PV406576–PV406664 (S2 gene), PV406665–PV406757 (S3 gene), and PV406758–PV406846 (S4 gene).

## Supplementary Materials

The following supporting information can be downloaded at https://www.mdpi.com/article/10.3390/v17070926/s1, Figure S1: Comparison of avian reovirus (ARV) qRT-PCR Ct values across different samples and purposes; Figure S2: Time-calibrated maximum likelihood phylogenetic tree reconstructed using the L1 (a), L2 (b), M1 (c), M3 (d), S2 (e), S3 (f), and S4 (g) sequences from this study; Figure S3. Maximum likelihood phylogenetic tree (a) of the full-length S1 gene. The two major clades are composed primarily of turkey hosts (b) and composed of turkey hosts and ruddy turnstones (c), respectively. Host species are color-coded. Figure S4: Reconstruction of ARV population dynamics using L3 as an example; Figure S5. Regression of root-to-tip genetic distance against sampling time from TempEst for all ten ARV genome segments. Table S1: Summary of metadata from the Iowa State University Veterinary Diagnostic Laboratory (ISU-VDL) in this study; Table S2: List of accessions and host species for each of the six clades of the M2 segment; Table S3: Analysis of selective pressure on the σC protein and M2 using the Mixed Effects Model of Evolution (MEME), Fixed Effects Likelihood (FEL), and Fast, Unconstrained Bayesian AppRoximation (FUBAR) methods.
