# Supplementary figures and images for: Investigation of Avian Reovirus Evolution and Cross-Species Transmission in Turkey Hosts by Segment-Based Temporal Analysis
Source: Viruses. 2025 Jun 28;17(7):926. doi: 10.3390/v17070926 (PMC12300447; doi:10.3390/v17070926)

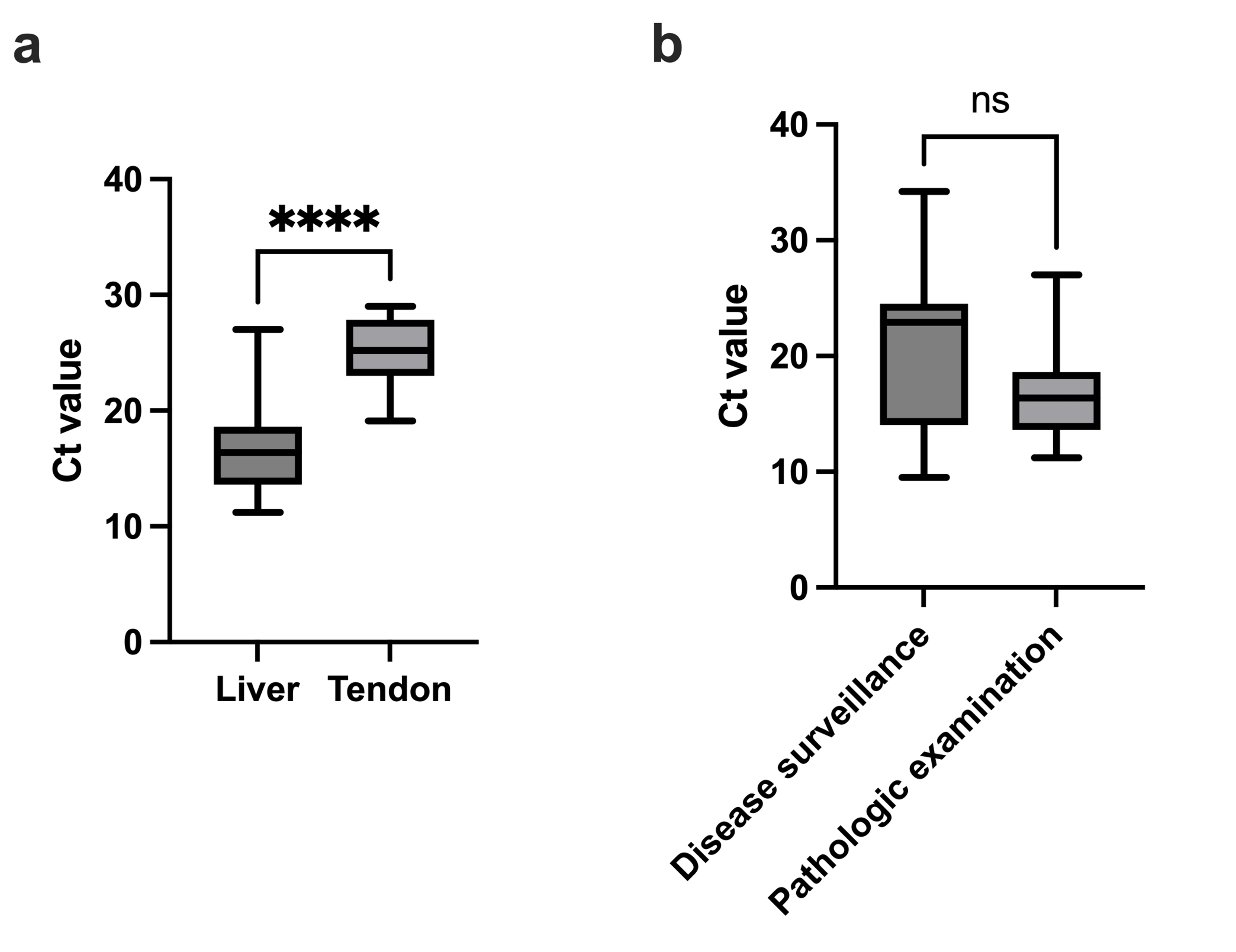

Supplement: Supplementary file 1 [file viruses-17-00926-s001.zip › Supplementary Figure S1.jpg]

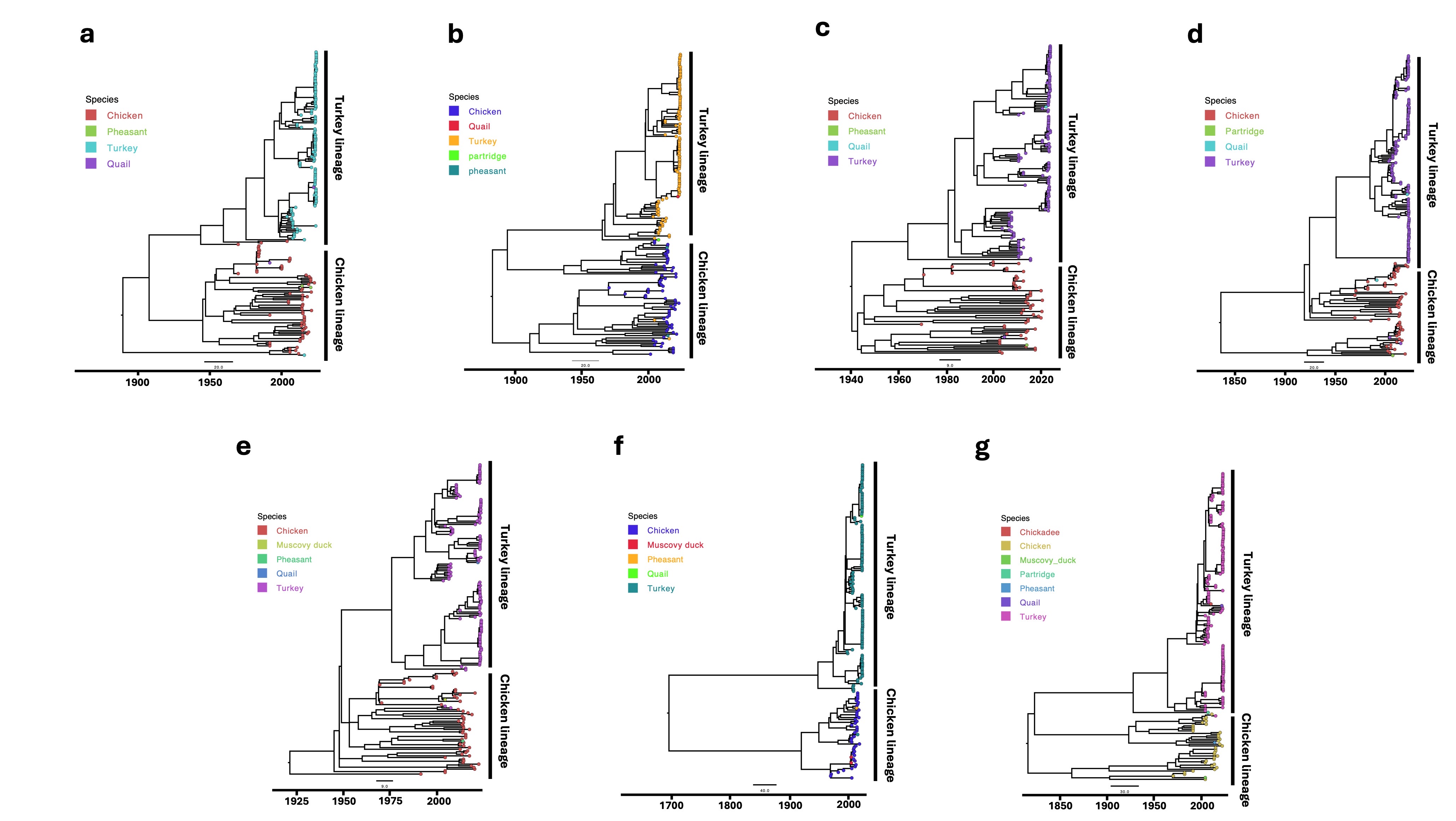

Supplement: Supplementary file 1 [file viruses-17-00926-s001.zip › Supplementary Figure S2.jpg]

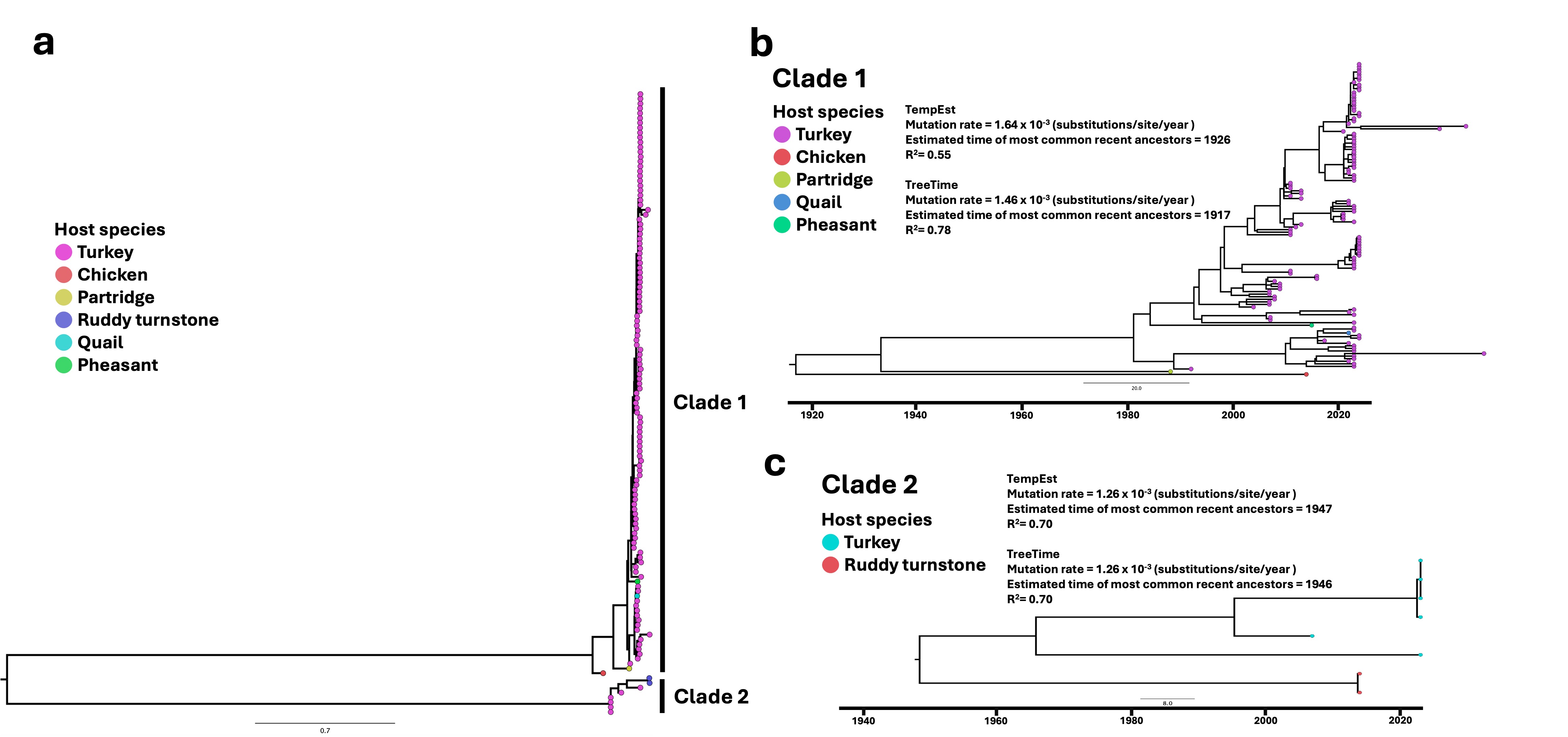

Supplement: Supplementary file 1 [file viruses-17-00926-s001.zip › Supplementary Figure S3.jpg]

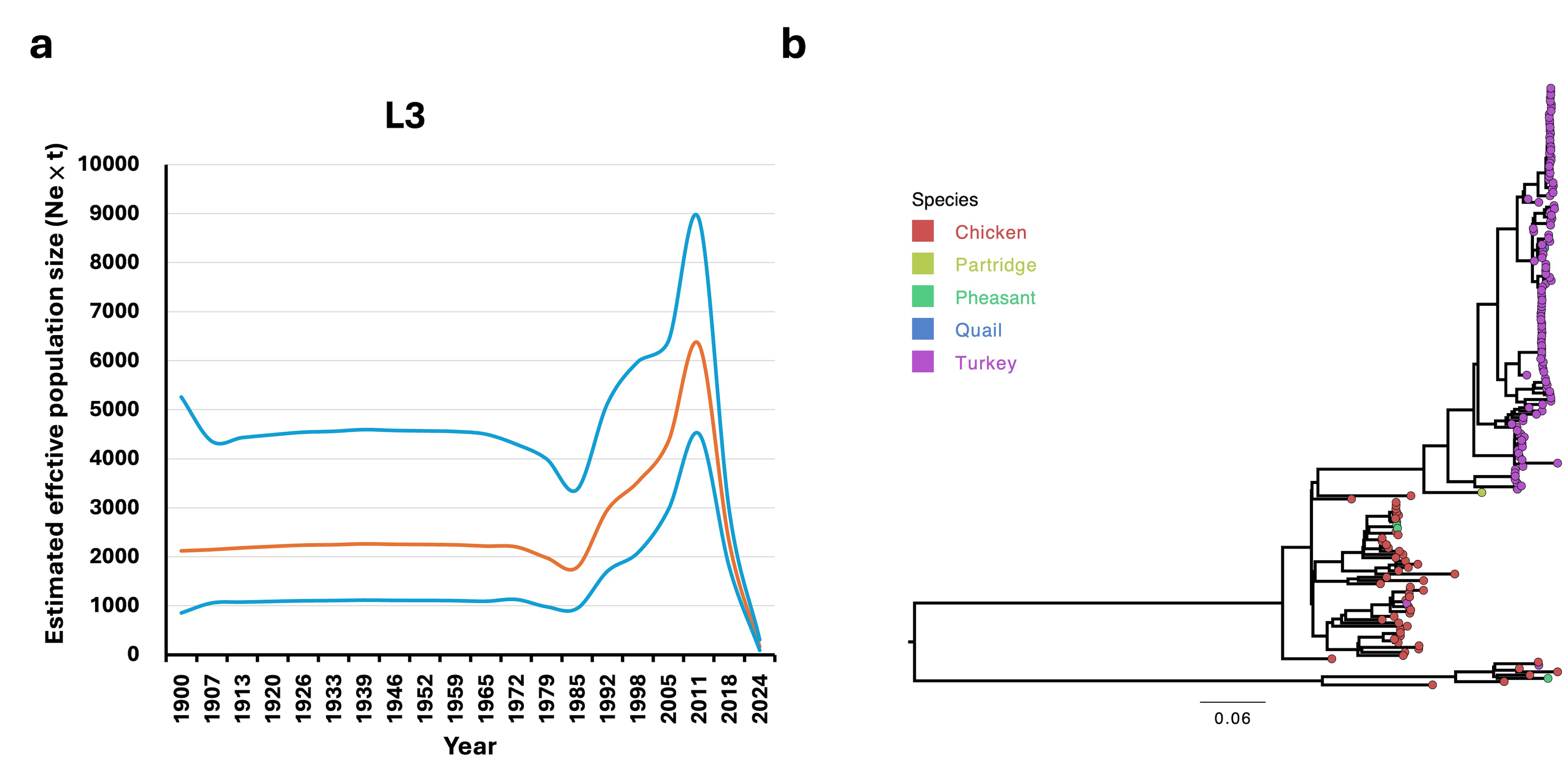

Supplement: Supplementary file 1 [file viruses-17-00926-s001.zip › Supplementary Figure S4.jpg]

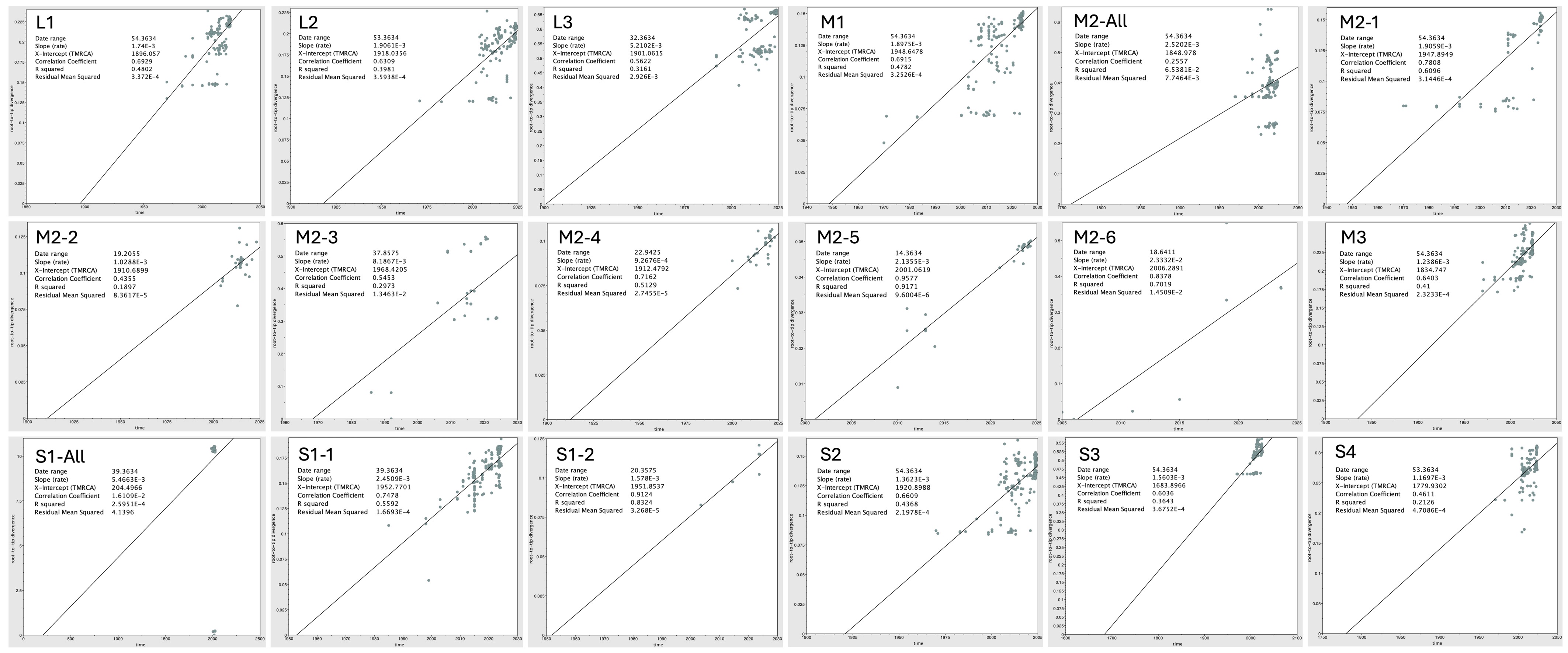

Supplement: Supplementary file 1 [file viruses-17-00926-s001.zip › Supplementary Figure S5.jpg]
